# Potential pharmaceuticals targeting neuroimmune interactions in treating acute lung injury

**DOI:** 10.1002/ctm2.1808

**Published:** 2024-08-11

**Authors:** Di Wu, Ximing Liao, Jing Gao, Yixuan Gao, Qiang Li, Wei Gao

**Affiliations:** ^1^ Department of Pulmonary and Critical Care Medicine Shanghai East Hospital School of Medicine Tongji University Shanghai P. R. China; ^2^ Department of Gynaecology Shandong Provincial Hospital Affiliated to Shandong First Medical University Jinan P. R. China

**Keywords:** acute lung injury, airway innervation, inflammation, neuroimmune interaction, potential pharmaceuticals

## Abstract

**Background and main body:**

Although interactions between the nervous and immune systems have been recognized decades ago, it has become increasingly appreciated that neuroimmune crosstalk is among the driving factors of multiple pulmonary inflammatory diseases including acute lung injury (ALI). Here, we review the current understanding of nerve innervations towards the lung and summarize how the neural regulation of immunity and inflammation participates in the onset and progression of several lung diseases, especially ALI. We then present advancements in the development of potential drugs for ALI targeting neuroimmune interactions, including cholinergic anti‐inflammatory pathway, sympathetic‐immune pathway, purinergic signalling, neuropeptides and renin‐angiotensin system at different stages from preclinical investigation to clinical trials, including the traditional Chinese medicine.

**Conclusion:**

This review highlights the importance of considering the therapeutic strategy of inflammatory diseases within a conceptual framework that integrates classical inflammatory cascade and neuroimmune circuits, so as to deepen the understanding of immune modulation and develop more sophisticated approaches to treat lung diseases represented by ALI.

**Key points:**

- The lungs present abundant nerve innervations.
- Neuroimmune interactions exert a modulatory effect in the onset and progression of lung inflammatory diseases, especially acute lung injury.
- The advancements of potential drugs for ALI targeting neuroimmune crosstalk at different stages from preclinical investigation to clinical trials are elaborated.
- Point out the direction for the development of neuroimmune pharmacology in the future.

## Graphical abstract

1. The lungs present abundant nerve innervations.

2. Neuroimmune interactions exert a modulatory effect in the onset and progression of lung inflammatory diseases, especially acute lung injury (ALI).

3. The advancements of potential drugs for ALI targeting neuroimmune crosstalk at different stages from preclinical investigation to clinical trials are elaborated.

4. Point out the direction for the development of neuroimmune pharmacology in the future.

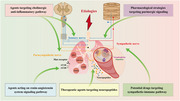

## 1 INTRODUCTION

Acute respiratory distress syndrome (ARDS) is thought to be a severe form of acute lung injury (ALI), which has an acute onset and rapid progression and is often characterized by diffuse lung inflammation and oedema.^1^ ALI or ARDS is caused by multiple predisposing conditions, which can be roughly divided into sepsis and non‐sepsis etiologies.^2^ The limited means of prevention and treatment contribute to high mortality and morbidity of the disorder. In the LUNG SAFE study^3^ concerning patients admitted to ICUs of 50 countries, 10.4% of the patients met ARDS criteria with hospital mortality rates of 34.9%, 40.3% and 46.1% for mild, moderate and severe ARDS, respectively. Given the geoeconomic variation, PRoVENT‐iMiC^4^ (international observational research of ICU patients from 10 Asian middle‐income countries) exhibited a lower incidence (7%) but higher mortality rate (45%) of ARDS compared to the LUNG SAFE study. Besides, another multicenter cohort study on patients surviving 2 years after ALI indicated a median cost of $35 259 for every survivor, which brought a significant economic burden to both the families and the healthcare system.^5^

The poor performance of ALI can be largely attributed to its complex pathophysiology, including overwhelming lung inflammation, increased alveolar permeability, as well as damaged alveolar epithelium and vascular endothelium, leading to decreased lung volume, reduced compliance and imbalance of ventilation/blood flow.^6^, ^7^, ^8^, ^9^ Despite great efforts made in developing novel pharmacotherapies for ALI,^10^ very few have shown clinical efficacy, which has troubled physicians for a long time. Actually, of all the potential interventions, only low tidal volume ventilation has been demonstrated to be beneficial for the patients.^11^ Therefore, discovering new pathogenesis and related therapeutic approaches to manage ALI is of great importance to a better clinical prognosis.

Recently, researchers have paid more attention to neuroimmune interactions, which serve as an underestimated novel mechanism in regulating excessive inflammatory responses of various disorders. The nerve system is capable of receiving stimuli and relaying signals in the form of neurotransmitters and/or neuropeptides to the adjacent immune cells via specific receptors, the cells can also release mediators, which in turn stimulate nearby endings of peripheral nerve.^12^ These close interactions between neurons and immune cells have been found to be a key mechanism driving the genesis of multiple inflammatory diseases.^13^, ^14^ In the development of ALI, immune cells are also innervated by neural activity,^15^, ^16^ and manipulation of neurotransmitter/neuropeptide or their receptors may become a new strategy to treat the syndrome. Accordingly, multiple agents have emerged to potently regulate the neuroimmune crosstalk, aiming to inhibit the disease‐associated inflammatory overactivation.

**FIGURE 1:**
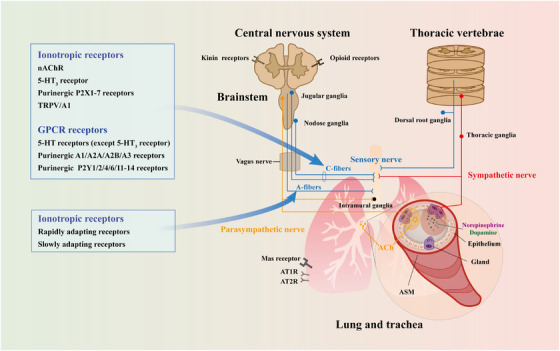
Overview of innervation in the lung. The lung is mainly innervated by sensory neurons arising from the jugular nodose complex or dorsal root ganglia (shown in blue), parasympathetic neurons originating from the brainstem or vagus nerve (shown in orange), and sympathetic neurons coming from the thoracic ganglia (shown in red). nAChR, nicotinic acetylcholine receptor; 5‐HT, 5‐ hydroxytryptamine; TRPV/A1, Transient receptor potential vanilloid 1/ ankyrin 1; GPCR, G‐protein‐coupled receptor; AT1R, angiotensin II type 1 receptor; AT2R, angiotensin II type 2 receptor; ACh, acetylcholine; ASM, airway smooth muscle.

**FIGURE 2:**
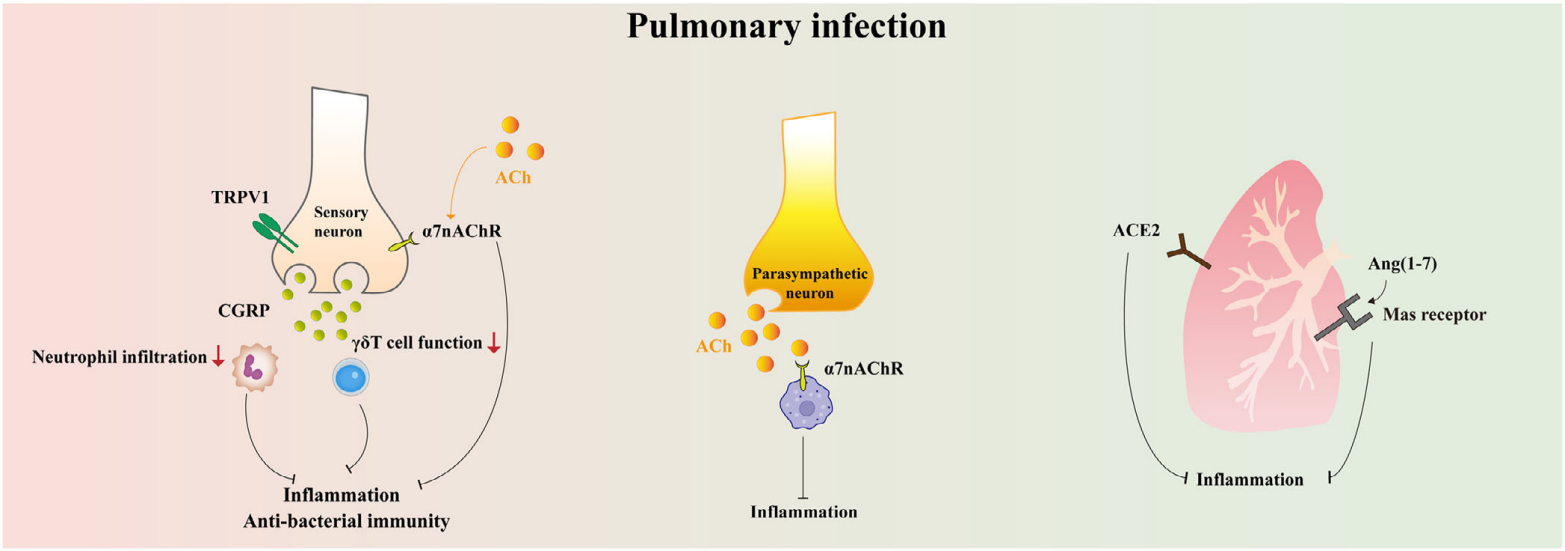
Neuroimmune interactions in pulmonary infection. The major pathways underlying the neuroimmune crosstalk during pulmonary infection. TRPV1, transient receptor potential vanilloid 1; α7nAChR, α7 nicotinic acetylcholine receptor; CGRP, calcitonin gene‐related product; ACh, acetylcholine; ACE2, angiotensin I‐converting enzyme 2; Ang (1‐7), angiotensin (1‐7).

**FIGURE 3:**
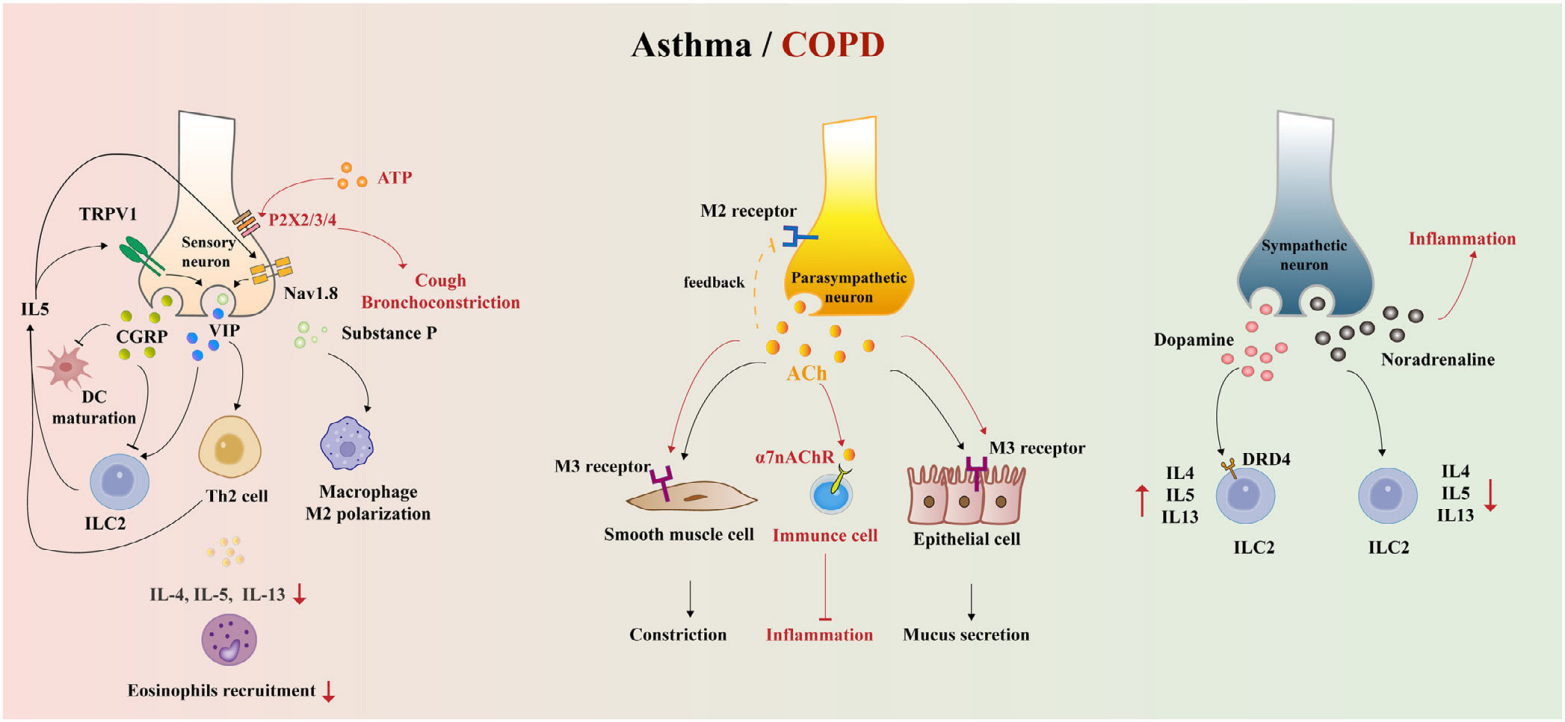
Neuroimmune interactions in asthma and COPD. The major pathways underlying the neuroimmune crosstalk during asthma and COPD. Black arrows represent neuroimmune communication pathways in asthma, while red arrows represent neuroimmune communication pathways in COPD. COPD, chronic pulmonary obstructive disease; TRPV1, transient receptor potential vanilloid 1; ATP, adenosine triphosphate; Nav 1.8, voltage‐gated sodium channel 1.8; IL, interleukin; CGRP, calcitonin gene‐related product; VIP, vasoactive intestinal peptide; DC, dendritic cell; ILC2, innate lymphoid cell 2; Th2 cell, T helper 2 cell; M2/M3 receptor, muscarinic acetylcholine (M)_2/3_ receptor; ACh, acetylcholine; α7nAChR, α7 nicotinic acetylcholine receptor; DRD4, dopamine receptor D4.

**FIGURE 4:**
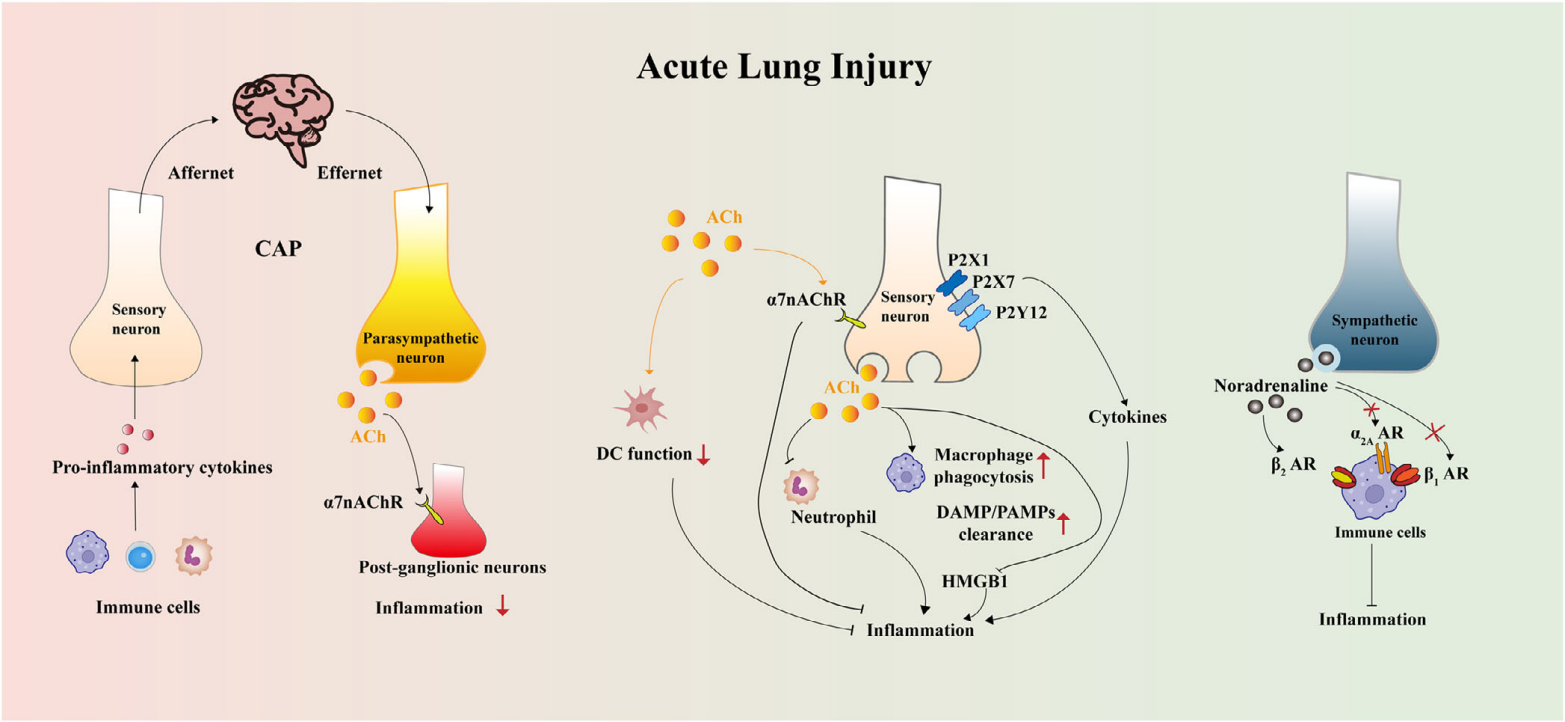
Neuroimmune interactions in ALI. The major pathways underlying the neuroimmune crosstalk during ALI. The major pathways underlying the neuroimmune crosstalk during ALI. ALI, acute lung injury; CAP, cholinergic anti‐inflammatory pathway; ACh, acetylcholine; α7nAChR, α7 nicotinic acetylcholine receptor; DC, dendritic cell; DAMP, damage‐associated molecular pattern; PAMP, pathogen‐associated molecular pattern; HMGB1, high mobility group box 1 protein; AR, adrenergic receptors.

**FIGURE 5:**
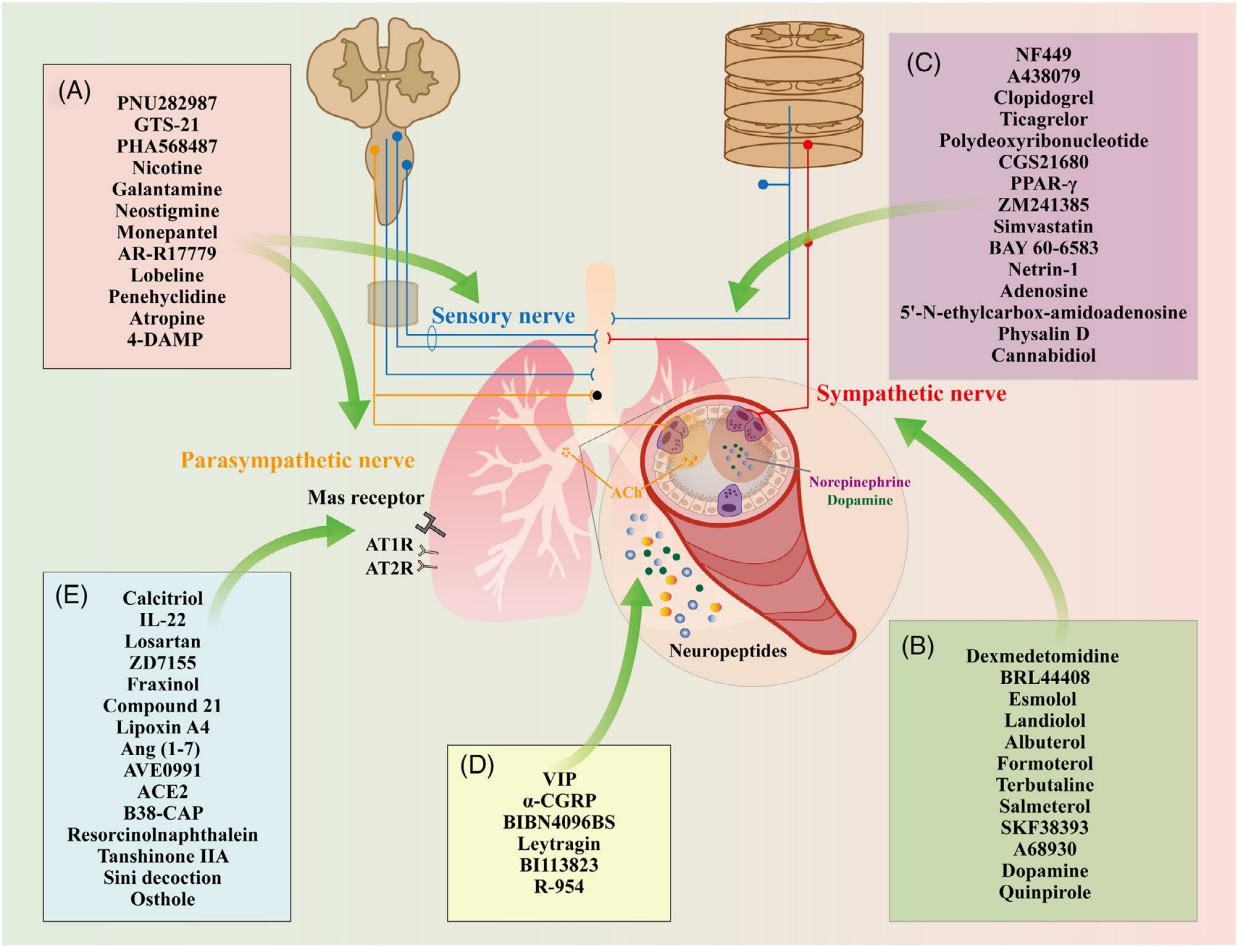
Potential drugs targeting neuroimmune interactions. (A–E) The neuroimmune modulation can be achieved through the following pathways: cholinergic anti‐inflammatory pathway (A), sympathetic‐immune pathway (B), purinergic signalling (C), neuropeptides (D) and renin‐angiotensin system (RAS) (E). VIP, vasoactive intestinal peptide; α‐CGRP, α‐calcitonin gene‐related product; PPAR‐γ, peroxisome proliferator‐activated receptor‐γ; Ang (1‐7), angiotensin (1‐7); ACE2, angiotensin I‐converting enzyme 2; AT1R, angiotensin II type 1 receptor; AT2R, angiotensin II type 2 receptor; IL‐22, interleukin‐22; ACh, acetylcholine.

In this review, we first summarize the anatomic nerve innervations towards the lung, mainly including afferent innervation, central nervous system (CNS), and efferent innervation (Figure 1). We then recapitulate how the neural regulation of immunity and inflammation participates in the onset and progression of several pulmonary diseases, especially ALI (Figures 2, 3, 4). In addition, we focus on the current knowledge of potential drugs targeting the cholinergic anti‐inflammatory pathway (CAP), sympathetic‐immune pathway, purinergic signalling, neuropeptides, and renin‐angiotensin system (RAS) at different stages from preclinical investigation to clinical trials, including the traditional Chinese medicine, as well as discuss their promising usages in treating ALI/ARDS (Figure 5 and Tables 1, 2, 3, 4, 5). This review deepens the understanding of immune modulation and develops more sophisticated approaches to treat lung diseases represented by ALI.

**TABLE 1:** The development status of drugs targeting cholinergic anti‐inflammatory pathway.

Drugs	Utility	Preclinical/Clinical study	Aetiology	Model	Dose	References
PNU282987	α7nAChR agonist	Preclinical	Intratracheal LPS	Murine model	10 mg/kg	Pinheiro et al., 2021
GTS‐21	α7nAChR agonist	Preclinical	Renal ischemia‐reperfusion	Murine model	4 mg/kg	Goto et al., 2022
		Preclinical	Mechanical ventilator	Murine model	8 mg/kg	Kox et al., 2011
PHA568487	α7nAChR agonist	Preclinical	Intratracheal LPS	Murine model	0.4 mg/kg	Su et al., 2010
Nicotine	α7nAChR agonist	Preclinical	Severe acute pancreatitis	Rat model	400 µg/kg	Ma et al., 2016
Galantamine	Acetylcholinesterase inhibitor	Preclinical	Intravenous LPS	Rat model	5 mg/kg	Li et al., 2016
Neostigmine	Acetylcholinesterase inhibitor	Preclinical	Intranasal LPS	Murine model	0.03/0.17 mg/kg	Zhang et al., 2022
Monepantel	nAChR agonist	Preclinical	Intranasal LPS	Murine model	5 mg/kg	Zhang et al., 2022
AR‐R17779	α7nAChR agonist	Preclinical	Intranasal LPS	Murine model	5 mg/kg	Zhang et al., 2022
Lobeline	nAChR agonist	Preclinical	Intranasal LPS	Murine model	0.33/1.67/5 mg/kg	Zhang et al., 2022
		Clinical	–	Sixteen patients	20 mg for 1 day	Zhang et al., 2022
Penehyclidine hydrochloride	M_3_ receptor antagonist	Preclinical	LPS	HPMVECs	2 µg/mL	Yuan et al., 2018
		Preclinical	Intravenous LPS	Rat model	0.3/1/3 mg/kg	Shen et al., 2009
Atropine	Muscarinic receptor antagonist	Preclinical	Intratracheal LPS	Murine model	3 mg/kg	Xu et al., 2012
4‐DAMP	M_3_ receptor antagonist	Preclinical	Intratracheal LPS	Murine model	3 mg/kg	Xu et al., 2012

Abbreviations: HPMVECs, human pulmonary microvascular endothelial cells; LPS, lipopolysaccharide; M_3_ receptor, muscarinic acetylcholine (M)_3_ receptor; nAChR, nicotinic acetylcholine receptor.

**TABLE 2:** The development status of agents targeting sympathetic‐immune pathway.

Drugs	Utility	Preclinical/Clinical study	Aetiology	Model	Dose	References
Dexmedetomidine	α_2_AR agonist	Preclinical	Intranasal IL‐17	Murine model; mouse primary epithelial cells	2.5/5 µg/mouse; 1 nM	Zhang et al., 2018
		Preclinical	Intravenous LPS	Rat model	100 µg/kg	Jiang et al., 2021
		Preclinical	Intravenous LPS	Rat model	0.5/2.5/5.0 µg/kg	Jiang et al., 2015
		Preclinical	Intraperitoneal LPS	Murine model	25 µg/kg	Zhang et al., 2018
		Preclinical	ALI rat serum	NR8383 cell line	0.1/1 µM	Chen et al., 2020
		Preclinical	Renal ischemia‐reperfusion	Murine model	25 µg/kg	Chen et al., 2016
		Preclinical	LPS	Human lung fibroblast cell line	0.1/1 nM	Zhang et al., 2018
		Preclinical	Intratracheal LPS	Murine model	40 µg/kg	Song et al., 2020
BRL44408	α_2A_AR antagonist	Preclinical	Cecal ligation and puncture	Rat model	5 mg/kg	Ji et al., 2012; Cong et al., 2020
Esmolol	β_1_AR antagonist	Preclinical	Acute pancreatitis	Rat model	15 mg/kg	Zhang et al., 2016
Landiolol	β_1_AR antagonist	Preclinical	Intraperitoneal LPS	Rat model	100 µg/kg	Matsuishi et al., 2016
Albuterol/ Formoterol	β_2_AR agonists	Preclinical	Intratracheal LPS or IgG immune complexes for murine models; LPS for cells	Murine model; MLE‐12 cell line	10^−6^ M	Bosmann et al., 2012
Terbutaline	β_2_AR agonist	Preclinical	Intratracheal pseudomonas aeruginosa	Rat model	10^−4^ M	Robriquet et al., 2011
Albuterol	β_2_AR agonist	Clinical	–	282 patients	5 mg every 4 hours for 10 days	Matthay et al., 2011; Lakshminarayana et al., 2012
Salmeterol	β_2_AR agonist	Clinical	–	179 patients	100 mg twice daily for 72 h	Perkins et al., 2011; Perkins et al., 2014
SKF38393	Dopamine D1/D5 receptor agonist	Preclinical	Intranasal LPS; LPS	Murine model; THP‐1/BEAS‐2B cell lines	5 mg/kg; 0.1/1/10/50 uM	Meng et al., 2023
A68930	Dopamine D1 receptor agonist	Preclinical	Spinal cord injury	Rat model	5 mg/kg	Jiang et al., 2016
Dopamine/Quinpirole	Dopamine/D2 receptor agonist	Preclinical	Intraperitoneal LPS	Murine model	50 mg/kg	Vohra et al., 2012

Abbreviations: AR, adrenergic receptors; BEAS‐2B cell line, human bronchial epithelial cell line; LPS, lipopolysaccharide; MLE‐12 cell line, mouse lung epithelial cell line; NR8383 cell line, rat alveolar macrophage cell line; THP‐1 cell line, human monocyte cell line.

**TABLE 3:** The development status of drugs targeting purinergic signalling.

Drugs	Utility	Preclinical/Clinical study	Aetiology	Model	Dose	References
NF449	P2X1 receptor antagonist	Preclinical	Intraperitoneal LPS + anti‐MHC I antibody	Murine model	10 mg/kg	El Mdawar et al., 2019
A438079	P2X7 receptor antagonist	Preclinical	Intratracheal LPS; LPS+ATP	Murine model; mouse BMDMs	80 mg/kg; 5 mM	Wang et al., 2015
Clopidogrel	P2Y12 receptor antagonist	Preclinical	Cecal ligation and puncture	Murine model	10/30 mg/kg	Liverani et al., 2016
Clopidogrel/ Ticagrelor	P2Y12 receptor antagonist	Preclinical	LPS	HPMVECs	20 µM	Han et al., 2022
Polydeoxyribonucleotide	A2A receptor agonist	Preclinical	Intratracheal LPS	Rat model	8 mg/kg	Ko et al., 2020
CGS21680	A2A receptor agonist	Preclinical	Intratracheal LPS + high oxygen	Murine model	125 µg/dose	Aggarwal et al., 2013
PPAR‐γ	A2A receptor agonist	Preclinical	Intravenous LPS	Murine model	3 mg/kg	He et al., 2013
ZM241385	A2A receptor antagonist	Preclinical	Severe TBI; LPS	Murine model; mouse BMDMs	1 mg/kg; 1 µM	Dai et al., 2013
Simvastatin	A2B receptor agonist	Preclinical	Intratracheal LPS	Rat model	20/40/80 mg/kg	Li et al., 2021
BAY 60−6583	A2B receptor agonist	Preclinical	Inhaled LPS	Murine model	2 mg/kg	Schingnitz et al., 2010
		Preclinical	Inhaled LPS; LPS	Murine model; PMNs, HMVEC/A549 cell lines	2 mg/kg; 0.1/1/10 ng/mL	Konrad et al., 2012
Netrin‐1	A2B receptor agonist	Preclinical	Intravenous LPS	Murine model	1 mg/mouse	He et al., 2014
Adenosine/5′‐N‐ethylcarbox‐amidoadenosine	Adenosine receptor agonist	Preclinical	Intratracheal LPS	Murine model	3.5 µM/mouse	Gonzales et al., 2014
Physalin D	P2X7 receptor antagonist	Preclinical	Intrathoracic ATP and LPS	Rat model; mouse peritoneal macrophages	0.001‐100 mg/kg for mice; 1/10/50/100/500 ng/mL	Arruda et al., 2021
Cannabidiol	A2A receptor agonist	Preclinical	Intranasal LPS	Murine model	20 mg/kg	Ribeiro et al., 2012

Abbreviations: A549 cell line, human lung adenocarcinoma cell line; ATP, adenosine triphosphate; BMDMs, bone marrow‐derived macrophages; HPMVECs, human pulmonary microvascular endothelial cells; LPS, lipopolysaccharide; MHC I, major histocompatibility complex I; PMNs, polymorphonuclear leukocytes; TBI, traumatic brain injury.

**TABLE 4:** The development status of agents targeting neuropeptides.

Drugs	Utility	Preclinical/Clinical study	Aetiology	Model	Dose	References
VIP	VIP receptor agonist	Preclinical	Intratracheal LPS	Murine model; mouse primary peritoneal macrophages	5×10^7^ transducing units/kg; 1/10/100 nM	Zhou et al., 2020
		Preclinical	Intraperitoneal LPS	RAW264.7 cell line	10^−10^/10^−9^/10^−8^ /10^−7^/10^−6^ M	Ran et al., 2015
α‐CGRP	CGRP receptor agonist	Preclinical	Intratracheal LPS	Rat model	0.4 µg/kg	Yang et al., 2015
		Preclinical	LPS	RAW264.7 cell line	1/10/100 nM	Duan et al., 2017
BIBN4096BS	CGRP receptor antagonist	Preclinical	Combined burn and smoke inhalation	Sheep model	32 µg/kg, followed by an infusion of 6.4 µg/kg/h for 48 h	Lange et al., 2009
Leytragin	δ‐opioid receptor agonist	Preclinical	Intratracheal LPS	Murine model	0.1 mg/kg	Karkischenko et al., 2021
BI113823	Kinin B1 receptor antagonist	Preclinical	Intratracheal LPS	Murine model	30 mg/kg	Nasseri et al., 2015
R‐954	Bradykinin B1 receptor antagonist	Preclinical	Intranasal LPS	Murine model	200 µg/kg	Campanholle et al., 2010

Abbreviations: ATP, adenosine triphosphate; CGRP, calcitonin gene‐related product; LPS, lipopolysaccharide; RAW264.7 cell line, mouse macrophage cell line; TBI, traumatic brain injury; VIP, vasoactive intestinal peptide.

**TABLE 5:** The development status of drugs targeting the renin‐angiotensin system.

Drugs	Utility	Preclinical/Clinical study	Aetiology	Model	Dose	References
Calcitriol	Vitamin D agonist	Preclinical	Intravenous LPS	Rat model	1/5/25 mg/kg	Xu et al., 2017
IL‐22	Ang II inhibitor	Preclinical	Subcutaneous Ang II	Murine model; rat PMVECs	20 µg/kg; 20 ng/mL	Wu et al., 2017
Losartan	AT1R antagonist	Preclinical	Intratracheal LPS	Murine model	10 mg/kg	Chen et al., 2013
ZD7155	AT1R antagonist	Preclinical	Intratracheal LPS	Rat model	1/10/20 mg/kg	Deng et al., 2012
Fraxinol	AT1R suppressor	Preclinical	Intratracheal LPS	Murine model; RAW264.7 cell line	20/40/80 mg/kg 5/10/25 µM	Wu et al., 2022
Compound 21	AT2R agonist	Preclinical	Intranasal LPS; LPS	Murine model; THP‐1 cell line	0.3 mg/kg 0.01/0.1/1/10 µM	Chen et al., 2024
Lipoxin A4	ACE2 agonist	Preclinical	Intraperitoneal LPS	Murine model	100 µg/kg	Chen et al., 2018
Ang (1‐7)	Short fragments of Ang II	Preclinical	Oleic acid infusion/ventilator/acid aspiration	Rat model; murine model	500 pmol/kg, 50 pmol/kg; 50 pmol/kg, 50 µg/mouse	Klein et al., 2013
AVE0991	Ang (1‐7) analog	Preclinical	Intravenous oleic acid	Rat model	500 pmol/kg	Klein et al., 2013
ACE2	Recombinant ACE2	Preclinical	Intratracheal SARS‐CoV‐2 spike RBD protein + LPS	Murine model	1 mg/kg	Zhang et al.,2022
B38‐CAP	ACE2‐like carboxypeptidase	Preclinical	Cecal ligation and puncture/acid aspiration	Murine model	2 mg/kg	Minato et al., 2022
		Preclinical	Intranasal/ intratracheal SARS CoV‐2	Hamster model/ murine model	2 mg/kg/day	Yamaguchi et al., 2021
Resorcinolnaphthalein	ACE2 activator	Preclinical	Intratracheal LPS	Murine model	15 ng/kg	Huang et al., 2020
Tanshinone IIA (danshen)	ACE2/Ang (1‐7) activator	Preclinical	Intratracheal paraquat	Rat model	25 mg/kg	Wang et al., 2018
Sini decoction	ACE2/Ang (1‐7) activator	Preclinical	Intraperitoneal LPS	Murine model; HUVECs	5 g/kg; 6.2/12.5/25/50 mg/mL	Chen et al., 2019
		Preclinical	Intratracheal E.coli	Murine model	5 g/kg	Liu et al., 2018
Osthole	ACE2/Ang (1‐7) activator	Preclinical	Intratracheal LPS	Murine model	40 mg/kg	Shi et al., 2013

Abbreviations: ACE2, angiotensin I‐converting enzyme 2; Ang, angiotensin; AT1R angiotensin II type 1 receptor; AT2R, angiotensin II type 2 receptor; HUVECs, human umbilical vein endothelial cells; LPS, lipopolysaccharide; PMVECs, pulmonary microvascular endothelial cells; RAW264.7 cell line, mouse macrophage cell line; SARS‐CoV‐2 spike RBD protein, severe acute respiratory syndrome coronavirus‐2 spike receptor‐binding domain protein; THP‐1 cell line, Human monocyte cell line.

## 2 NERVE INNERVATION TOWARDS THE LUNG

The nerve system is categorized as CNS including the spinal cord and brain, and the peripheral nervous system, which dominates the lung and is broadly divided into the afferent sensory nerve system and the efferent nerve system. Within the latter, the autonomic nerve is located in the viscus and controls smooth muscles, cardiac muscles, and glands. It can be further classified into parasympathetic and sympathetic nerves according to their functional features (Figure 1).

### 2.1 Afferent innervation

The afferent nerves of the lung and respiratory tract are mainly the afferent fibers of the vagus nerve (about 20%), which communicate the external and internal environments to the CNS. Most of the afferent nerve cell bodies are located in nodose or jugular vagal ganglia, while a small proportion of afferent nerves come from the thoracic dorsal root ganglia. Vagal sensory fibers are classified into fastest Aβ‐fibers, intermediate Aδ‐fibers and slowest unmyelinated C‐fibers according to their conduction velocities. A‐fibers are often activated by mechanical forces such as stretch and touch, but they are not sensitive to chemical stimuli. On the contrary, C‐fibers can be activated by chemical, thermal and physical stimuli. In addition, Aβ‐fiber mechanoreceptors are subcategorized as rapidly and slowly adapting receptors based on the action potential adaptation. Any circumstances altering the balance of mechanical forces, such as bronchoconstriction resulting from oedema or mucus overproduction, can lead to the activation of the two receptors.^17^ A long list of chemicals causes a mechanical action on airway structural cells, followed by indirect sensitization of the receptors. Yet, adenosine triphosphate (ATP) itself can directly activate the intrapulmonary mechanoreceptors through P2X2/3 purinergic receptors on most nodose neurons.^18^, ^19^

Unmyelinated C‐fibers outnumber A‐fibers at a ratio of around 8:1 in the afferent nerves of the airway,^20^ and are subclassified as nodose and jugular C‐fibers according to their locations. Nodose C‐fibers are responsive to adenosine, ATP and 5‐hydrotryptamine, whereas jugular C‐fibers express substance P and calcitonin gene‐related product (CGRP), which directly induce the contraction of airway smooth muscle (ASM).^21^ Besides, C‐fibers express a spectrum of receptors, mainly including ligand/voltage‐gated ion channels and G‐protein‐coupled receptors (GPCRs). Among ionotropic receptors, the well‐studied nicotinic cholinergic receptor (nAChR) is a pentamer made up of 17 subunits α1‐10, β1‐4, γ, δ and ε, spanning the membrane four times.^22^ Interestingly, this type of receptor is unlikely to be stimulated by endogenous acetylcholine (ACh) from postganglionic cholinergic nerves but is responsive to epithelial‐derived ACh.^23^ As for the 5‐hydrotryptamine receptor family, all members belong to GPCRs except for the 5‐hydrotryptamine‐3 receptor, which is an ionotropic receptor that is selectively expressed in nodose sensory neurons,^24^ and its activation leads to C‐fibers action potential discharge.^25^, ^26^ Purine receptors are divided into P1 (adenosine‐activated) and P2 (ATP or adenosine diphosphate‐activated) types.^27^ P1 receptors belong to the GPCR family and can be further categorized as A1, A2A, A2B and A3 subclasses, among which A2B is expressed in the lung (mainly in pulmonary epithelia, vascular endothelia and inflammatory cells).^28^, ^29^, ^30^, ^31^ P2 receptors include two main families, namely, P2X (ligand‐gated ion channels) with seven subtypes (P2X1–7) and P2Y (GPCRs) also with 7 subtypes (P2Y1, 2, 4, 6 and 11–14).^32^ ATP depolarizes all nodose neurons via the heteromeric channel P2X2/P2X3, but not in C‐fibers of jugular neurons, on which P2X2 expression is absent.^19^, ^33^

### 2.2 Central nervous system

The brainstem is the destination of all afferent sensory nerves within vagus nerves, and also the place where afferent nerves innervate the second‐order neurons, ascending to the higher brain region, descending to the spinal cord and projecting to adjacent brain stem nuclei. The embryological difference between nodose and jugular C‐fibers is also reflected in the destinations with the former tending to the medullary region in the nucleus of the solitary tract and adjacent postrema area, while the latter favouring the trigeminal nucleus area.^34^, ^35^ C‐fibers and most A‐fibers terminating in the nucleus of the solitary tract depend on non‐N‐methyl‐D‐aspartate receptors to complete the glutamatergic transmission, whereas a small portion of Aδ‐fibers requires N‐methyl‐D‐aspartate receptors.^36^ However, current knowledge about neurons in the solitary tract area remains limited, let alone neurons in the trigeminal nucleus area.

Kinin receptors B1 and B2 belong to the GPCR family and are expressed in CNS^37^; besides, the B1 receptor is normally absent but rapidly and highly upregulated under inflammatory circumstances.^38^ Another member of GPCRs located in the brain is the opioid receptors, which are mainly classified into three classic types: µ, δ and κ. The intriguing receptors can be activated by both endogenous opioid peptides and exogenous opiate compounds.^39^

The RAS comprises the angiotensin I‐converting enzyme (ACE)‐angiotensin (Ang) II‐angiotensin receptor (ATR) axis and the ACE2‐Ang (1‐7)‐Mas receptor axis and is well known for its bidirectional role in the cardiovascular and urinary systems. All Ang peptides are derived from the cleavage of angiotensinogen in CNS, with the majority being produced by astrocytes and constitutively secreted into the interstitial space and cerebrospinal fluid.^40^ The angiotensinogen is then decomposed into Ang I by aspartyl‐protease rennin and further turned into active Ang II by ACE.^41^ The function of Ang II depends on its binding to distinct receptors, AT1R and AT2R, which are seven transmembrane GPCRs.^42^ Specifically, the two receptors mediate opposite effects: AT1R plays a significant role in inflammation, fibrosis, proliferation and vasoconstriction,^43^ while AT2R promotes anti‐inflammation, antifibrosis and vasodilation.^44^ Additionally, ACE2 is a homolog of ACE and cleaves Ang II into Ang (1‐7), which exerts lung protective actions via its Mas receptor.^45^

### 2.3 Efferent innervation

#### 2.3.1 Parasympathetic nerve

Most of the cholinergic parasympathetic nerves controlling tracheal walls arise from the ambiguous nucleus of the medulla and travel within the vagus nerves to intramural ganglia within airways,^46^ while a smaller part comes from the dorsal motor nucleus of the vagus nerves.^47^ After arriving at the intramural ganglia, parasympathetic nerves project to postganglionic neurons and send information to airway effector cells by releasing ACh.^48^

#### 2.3.2 Sympathetic nerve

The primary cell bodies of preganglionic sympathetic neurons are located in the upper six thoracic segments of the spinal cord, followed by projecting to secondary neurons in paravertebral sympathetic chain ganglia. The postganglionic sympathetic neurons originating from stellate and sympathetic chain ganglia dominate the lung, while those derived from stellate and superior cervical ganglia innervate the trachea.^49^ The termination of sympathetic nerve fibres is close to blood vessels, submucosal glands and ASM,^50^, ^51^ whose typical catecholamine neuromediators include noradrenaline and dopamine.

## 3 NEUROIMMUNE CROSSTALK IN LUNG DISEASES

### 3.1 Pulmonary infection

Neuroimmune interactions regulate innate immune defence within the lung, and whether the effect is beneficial or harmful depends on distinct physiological and pathological situations. In *Staphylococcus aureus* pneumonia, transient receptor potential vanilloid 1 (TRPV1)‐expressing sensory neurons suppress neutrophils infiltration into the lung and γδT cell effector functions, revealing a weakened antibacterial immunity. Otherwise, ablating the neurons or blocking CGRP can improve the overall survival of mice against lethal *S. aureus* infection.^52^ The expressions of TRPV1 and transient receptor potential ankyrin 1 in sensory neurons are enhanced shortly after rhinovirus infection, accompanied by elevated cough reflex sensitivity.^53^ In contrast, α7nAChR activation on bone‐marrow‐derived macrophage alleviates lung inflammation in influenza‐exposed mice.^54^ Vagal sensory neurons within airways also respond to influenza and express antiviral or pro‐inflammatory genes, which is accompanied by an increased leukocyte migration into the vagal ganglia.^55^ As for efferent innervation, the influenza virus activates the sympathetic nerves in the mouse lung, and peripheral sympathectomy improves the survival rate and restrains excess inflammation in the mice.^56^ Furthermore, the RAS plays a controversial role in the pathogenesis of ALI triggered by severe acute respiratory syndrome coronavirus 2 (SARS‐CoV‐2), which recognizes ACE2 on the cell surface via a spike binding domain, followed by membrane fusion and virus uptake.^57^ Experimental data indicate that the increased ACE2 expression by RAS blockers may facilitate viral cell entry.^58^ Conversely, nuclear ACE2 inhibitor reduces viral replication and pulmonary inflammation in SARS‐CoV‐2‐infected hamsters.^59^ On the other hand, ACE2 is a critical counter‐regulator of the RAS and may exert lung protective effects in COVID‐19. The above findings emphasize the necessity of solid evidence‐based data in future research. Besides, the oestrogen‐protected endothelial integrity through activating Ang (1‐7)/Mas receptor signalling probably explains the sex differences in mobility and mortality during the SARS‐CoV‐2 attack.^60^ (Figure 2)

### 3.2 Bronchial asthma

Bronchial asthma (or asthma) is orchestrated by various inflammatory cells and airway structural cells, including innate lymphoid cell (ILC)2, eosinophil, dendritic cells, as well as epithelial cells and ASM cells. Increasing evidence has linked the nervous system to inflammatory responses in asthma. Sensory neurons expressing TRPV1 and/or voltage‐gated sodium channel Nav1.8 produce neuropeptides such as CGRP, vasoactive intestinal peptide (VIP) and substance P, which participate in the formation of asthma.^61^ CGRP is a negative regulator of ILC2‐mediated allergic inflammation via its receptor subunits Calcrl and Ramp1.^62^ CGRP suppresses interleukin (IL)‐33‐induced ILC2 proliferation and subsequent eosinophil recruitment into the lung, thus protecting against tissue damage in allergic asthma models.^63^ CGRP also inhibits the maturation and function of dendritic cells, as well as regulates adaptive immune response in asthma.^64^ In ovalbumin or house dust mite‐challenged asthmatic mice, IL‐5 overproduction by the activated immune cells acts on TRPV1 and Nav1.8 to secret VIP, which stimulates resident ILC2s and T helper 2 cells, creating a positive feedback loop that aggravates allergic lung inflammation.^65^, ^66^ Additionally, asthma patients and animal models exhibit enhanced cholinergic innervation compared with healthy individuals. Mechanistically, ACh is known to induce mucus hypersecretion and bronchoconstriction through muscarinic ACh receptor (M)_3_ on airway epithelial cells and ASM cells, while the M_2_ receptor provides negative feedback on ACh secretion.^67^ As for the sympathetic nerve system, tyrosine hydroxylase‐expressing neurons activate β_2_ adrenergic receptor (AR) by noradrenaline secretion to restrain ILC2‐mediated inflammation.^68^ Besides, the postnatal transformation of the dopaminergic nerve to the adrenergic nerve is reported to be associated with asthma susceptibility in childhood through dopamine receptor D4^69^ (Figure 3).

### 3.3 Chronic pulmonary obstructive disease

As another common chronic respiratory disease, chronic pulmonary obstructive disease (COPD) is a major worldwide health problem characterized by irreversible airflow limitation and abnormal inflammatory response in the airway. Overactivated ATP signalling through P2X2/3/4 receptors in sensory neurons is observed in COPD animal models and related to pro‐inflammatory mediators generation, cough and bronchoconstriction.^70^, ^71^, ^72^ Comparatively, α7nAChR activation in immune cells exhibits modulatory actions in COPD by limiting the production of inflammatory markers linked to disease severity.^73^ The role of VIP in COPD is controversial, to be specific, the elevated serum VIP level has been identified to be associated with COPD exacerbation, but inhaled VIP is beneficial for life quality in COPD patients.^74^, ^75^ Besides, researchers also report the increased sputum CGRP and substance P levels in COPD patients, but the underlying mechanisms are largely obscure.^76^, ^77^ Parasympathetic release of ACh via vagus nerves has been well known to activate M_3_ muscarinic receptor, thus inducing bronchoconstriction,^78^ whereas M_3_ antagonist serves as a regular bronchodilator drug for COPD patients.^79^ Increased muscle sympathetic nerve activity caused by long‐term hypoxia is another characteristic of COPD and often accompanied by systemic inflammation, with the mechanisms unexplored.^80^ Interestingly, despite the attenuation of hyperinflation, the long‐acting inhaled β‐agonist does not appear to affect sympathoexcitation^81^ (Figure 3).

### 3.4 Acute lung injury

The pulmonary nervous system also plays a critical role in modulating acute inflammation of ALI. Pro‐inflammatory macrophage‐derived cytokine storms in ALI can be read by sensory neurons within vagus nerves and the signals are sent to the brain^82^; subsequently, efferent parasympathetic neurons release ACh to stimulate α7nAChR on post‐ganglionic neurons.^83^ The efferent fibres of vagus nerves are connected to the splenic nerves in the abdominal mesenteric ganglia, transmitting anti‐inflammatory signals to the spleen. The splenic nerve endings release noradrenaline, which then stimulates specific T lymphocytes expressing choline acetyltransferase to synthesize ACh. Thereafter, ACh inhibits inflammatory factor production by activating the α7nAChR in immune cells.^84^ This process is known as the CAP. Commonly, stimulation of vagus or splenic nerves exerts an anti‐inflammatory effect on lipopolysaccharide (LPS)‐induced immune cells; while vagotomy aggravates the inflammation.^83^ In the ventilator‐induced ALI rat model, pharmacological or electrical stimulation of vagus nerves ameliorates pulmonary injury via the α7nAChR‐dependent pathway.^85^ Similarly, α7nAChR agonist can attenuate hyperoxia‐induced ALI via alleviating the accumulation of high mobility group box 1 protein (HMGB1) in airways and circulation.^16^ In addition, blockage of P2 receptor P2X1, P2X7 or P2Y12 on C‐fibers is demonstrated to dampen excess pro‐inflammatory cytokines expression in different ALI models.^86^, ^87^, ^88^ The effect of neuroregulation on ALI is sometimes complicated since either activation (α_2_AR and β_2_AR) or inhibition (α_2A_AR and β_1_AR) of ARs can alleviate acute airway inflammation in the ALI animal models.^89^, ^90^, ^91^, ^92^ Besides, some studies have identified the protective roles of exogenous neuropeptides VIP and CGRP in improving lung damage in LPS‐induced ALI,^93^, ^94^, ^95^ while others have illustrated that CGRP antagonist decreases vascular permeability caused by LPS.^96^, ^97^ This may be attributed to the complex pathological process of ALI, and whether one strategy takes precedence over the other may quite depend on the specific situation in the disorder (Figure 4).

## 4 POTENTIAL PHARMACEUTICALS TARGETING NEUROIMMUNE PATHWAYS FOR ALI THERAPY

### 4.1 Therapeutic agents targeting the CAP

The CAP plays a crucial role in regulating inflammation in ALI/ARDS. The LPS‐induced inflammatory markers of ALI show significantly higher levels in cholinergic‐deficient than wild‐type mice, while selective α7nAChR agonist **PNU282987** can decrease neutrophil accumulation, IL‐1β and chemokine (C‐X‐C motif) ligand‐1 levels in the lung of ALI mice.^98^ As another agonist towards α7nAChR, **GTS‐21** reduces neutrophilic inflammation in renal ischemia‐reperfusion‐triggered ALI mice; splenectomy or splenic macrophage depletion limits its protective effect, indicating engagement of these cells in CAP.^99^ In the mechanical ventilator‐challenged mouse model, GTS‐21 attenuates the lung and plasma levels of tumour necrosis factor (TNF)‐α, as well as lung injury. However, modulating endogenous cholinergic signalling by mecamylamine and neostigmine can not affect the inflammatory response, implying that selective CAP stimulation may offer a new therapeutic strategy for this type of ALI.^100^ **PHA568487** and **nicotine**, the selective and nonselective α7nAChR agonists, both alleviate pulmonary damage in gram‐negative sepsis or severe acute pancreatitis‐associated ALI.^101^, ^102^ HMGB1 serves as a pivotal upstream regulator of inflammatory responses and correlate significantly with the severity in multiple diseases like ALI. A central acetylcholinesterase inhibitor **Galantamine**, as well as GTS‐21, are able to protect against ALI in animal models by attenuating HMGB1 accumulation in lung tissues.^103^, ^104^ Recently, Zhang et al. comprehensively investigated the protective role of the CAP pathway in the ALI model: the nAChR agonists **monepantel** and **lobeline**, the specific α7nAChR agonists **AR‐R17779** hydrochloride and GTS‐21, as well as the anticholinesterase **neostigmine** attenuated LPS‐induced pulmonary inflammation and tissue injury in ALI mice. Moreover, the authors conducted a pilot, nonrandomized, open‐label and controlled clinical trial of **lobeline** in the management of ALI/ARDS, which exhibited higher ventilator‐free days and ICU survival rate, along with decreased levels of inflammatory biomarkers in bronchoalveolar lavage fluid (BALF) and blood samples from patients within the lobeline group.^105^ Thus, nAChR agonist appears to be a potential therapeutic target for ALI.

Muscarinic M_3_ receptor has been shown to participate in LPS‐mediated pulmonary microvascular endothelial injury, which could be improved by the anticholinergic **penehyclidine hydrochloride** through the blockage of the M_3_ receptor.^106^, ^107^ Besides, non‐selective muscarinic cholinergic receptor antagonist **atropine** or selective M_3_ antagonist **4‐DAMP**, rather than M_1_ or M_2_ antagonist, shows regulatory effects on neutrophil infiltration, microvascular permeability and cytokines secretion in the lung of ALI mice, indicating the important role of M_3_ receptor in LPS‐stimulated pulmonary inflammation^108^ (Figure 5A and Table 1).

### 4.2 Potential drugs targeting sympathetic‐immune pathway

**Dexmedetomidine**, a highly selective α_2_AR agonist with analgesic and sedative effects, is one of the most well‐known drugs for clinical therapy of ARDS. It has also been well studied in the pathogenesis and treatment of ALI and is demonstrated to modulate the immuno‐inflammatory response through multiple mechanisms, thereby alleviating pulmonary microvascular hyperpermeability and lung tissue damage.^109^, ^110^, ^111^, ^112^, ^113^, ^114^, ^115^, ^116^ **BRL44408 maleate** is a specific α_2A_AR antagonist and has been validated to improve sepsis‐associated ALI by suppressing pro‐inflammatory mitogen‐activated protein kinase (MAPK) and nuclear factor‐κB (NF‐κB) pathways,^89^ as well as by downregulating the HMGB1 level.^117^ **Esmolol** is a selective β_1_AR blocker applied for various types of tachycardia. Interestingly, it also abates the increases in TNF‐α, IL‐6 and myeloperoxidase activity in BALF samples or lung tissues, thereby reducing disease severity and improving survival time in acute pancreatitis‐related ALI rats.^90^ Similarly, **landiolol hydrochloride** also exhibits therapeutic effects on the LPS‐challenged ALI model by decreasing pulmonary TNF‐α, IL‐6 and endothelin‐1.^91^

The β_2_AR agonists are the most widely prescribed medication for chronic airway diseases like COPD and asthma. Recently, they have also been extensively investigated in experimental and clinical ALI. Among them, **albuterol** and **formoterol** confine excess lung inflammation by inhibiting the c‐Jun N‐terminal kinase/MAPK pathway, while **terbutaline** improves alveolar‐capillary barrier function and enhances alveolar fluid clearance in ALI animal models.^92^, ^118^ To determine whether an β_2_AR agonist would improve clinical outcomes in patients with ALI, a multi‐centre, phase III randomized, placebo‐controlled clinical trial was conducted among the patients receiving aerosolized **albuterol** or saline every 4 h for up to 10 days. However, by measuring the ventilator‐free days as a primary outcome variable, the researchers concluded that routine use of β_2_AR agonists did not improve the prognosis in mechanically ventilated ALI patients (NCT 00434993).^119^, ^120^ In another randomized placebo‐controlled trial conducted at 12 UK centres, patients undergoing elective esophagectomy were randomly allocated to inhaled **salmeterol** or matched placebo treatment. The results showed that salmeterol administration did not prevent early ALI or improve organ failure, survival, or health‐related quality of life, though it did downregulate biomarkers of airway inflammation and epithelial injury in these patients.^121^, ^122^

In the peripheral dopaminergic nervous system, **SKF38393** is a selective agonist for dopamine D1/D5 receptors. Recently, we found that SKF38393 administration inhibited excessive inflammation and oxidative stress in macrophages, as well as maintained airway epithelial barrier function in LPS‐stimulated ALI mice, partly via the activation of nuclear factor erythroid‐2‐related factor 2 antioxidative system.^123^ Another D1 receptor agonist **A68930** is also demonstrated to exhibit therapeutic efficacy on spinal cord injury‐induced ALI rats by dampening the activation of NOD‐like receptor thermal protein domain associated protein 3 (NLRP3) signaling.^124^ In addition, pretreatment with **dopamine** or D2 receptor‐specific agonist **quinpirole** significantly increases survival rates and reduces neutrophil recruitment and pulmonary edema of LPS‐challenged mice. However, the effects are not observed in D2 receptor knockout mice, suggesting its role in dopamine‐mediated barrier protection.^125^ Conversely, as a dopaminergic antagonist, domperidone aggravates LPS‐induced TNF‐α and IL‐6 production in BALF of ALI mice, which may exacerbate the subsequent inflammatory injury^126^ (Figure 5B and Table 2).

### 4.3 Pharmacological strategies targeting purinergic signalling

Transfusion‐related ALI is a critical post‐transfusion respiratory syndrome, but the underlying biological responses remain unclarified. The ATP‐gated P2X1 cation channel is reported to participate in the development of the syndrome, with its selective antagonist **NF449** improving overall survival, reducing BALF protein leakage and lung interstitial oedema in the mouse model by targeting monocytes/macrophages.^87^ Likewise, the P2X7 blockers **A438079** suppresses LPS‐stimulated pro‐inflammatory cytokines release, neutrophil accumulation, and cysteinyl aspartate specific proteinase 1 activation by NLRP3 inflammasome in the ALI mouse model.^88^ **Physalin D** is extracted from *Physalis angulata* L. leaves and is capable of reversing ATP/LPS‐induced lung injury in mice through inhibiting P2X7 receptor function.^127^ Increasing studies have indicated an essential role of platelets in ALI pathogenesis; P2Y12 antagonist **clopidogrel** treatment or *P2Y12* knockout diminishes platelet activation, platelet‐leukocyte aggregates and subsequent lung damage in mice induced by sepsis.^128^ Consistently, P2Y12 inhibitors clopidogrel and **ticagrelor** are also able to reduce the inflammatory response, as well as improve the migration, function and permeability of endothelial cells in the LPS‐challenged cell model.^86^

Generally, activation of the adenosine A2A receptor is considered to be anti‐inflammatory. As an A2A receptor agonist, **polydeoxyribonucleotide** downregulates the production of LPS‐elevated pro‐inflammatory cytokines and apoptotic factors, and promotes the recovery of injured lung of ALI rats via potently attenuating MAPK/NF‐κB signaling pathway; meanwhile, the A2A receptor blocker 7‐dimethyl‐1‐propargylxanthine reverses the effects.^129^ **CGS21680 hydrochloride** is also an A2A receptor‐specific agonist and is confirmed to abrogate macrophage activation and histologic lung injury in LPS plus high oxygen‐exposed mice.^130^ Cannabidiol is a non‐psychotropic plant‐derived cannabinoid with potent immunomodulatory properties. **Cannabidiol** administration decreased leukocyte migration, alveolar protein leakage, pro‐inflammatory cytokines generation, and tissue damage in the LPS‐stimulated ALI mouse model. The underlying mechanism may be associated with the activation of the A2A receptor since a selective A2A antagonist ZM241385 can abrogate the protective effect of Cannabidiol.^131^ In addition, **peroxisome proliferator‐activated receptor‐γ** has been reported to upregulate A2A receptor expression in the ALI mouse lungs via binding to a DR10 response element within its premotor region. Hence, the combination of peroxisome proliferator‐activated receptor‐γ and A2A agonists is found to be a more efficient therapeutic strategy for ALI.^132^ Nevertheless, in a neurogenic ALI mouse model caused by severe traumatic brain injury (TBI), A2A receptor agonist CGS21680 exacerbates, whereas the inhibitor **ZM241385** mitigates the inflammatory damage within lung tissues, which is attributed to the elevated plasma glutamate after severe TBI.^133^, ^134^ The results indicate that when targeting the A2A receptor, full consideration should be given to whether it is nonneurogenic or neurogenic ALI; besides, combined therapy targeting both A2A and blood glutamate might be a potential strategy for TBI‐ALI management.

The adenosine A2B receptor may also be a potential therapeutic target for ALI/ARDS. In an ALI model caused by LPS, **simvastatin** protects rats from severe pulmonary damage by activating the A2B receptor; whereas, treatment with its antagonist PSB1115 counters the effect of simvastatin.^135^ Another A2B agonist **BAY 60−6583** is shown to decrease microvascular permeability and neutrophil migration into the pulmonary interstitium,^136^ thus attenuating lung inflammation and edema in ALI mouse models.^137^ However, in the trauma‐hemorrhagic shock‐induced rat model, BAY 60−6583 can reduce pulmonary permeability but fails to alleviate neutrophil infiltration and inflammation in the lung.^138^ A newly found anti‐inflammatory factor, **netrin‐1** dampens pulmonary inflammation and enhances alveolar fluid clearance to alleviate pulmonary edema in LPS‐challenged ALI through the activation of the A2B receptor. The beneficial effect of netrin‐1 is abolished by specific A2B receptor inhibitor PSB1115.^139^ Coherently, A2B receptor knockout mice show augmented mortality associated with excess inflammatory response upon LPS stimulation compared to wild‐type controls.^140^ Furthermore, the activation of adenosine receptors either by **adenosine** or **5′‐N‐ethylcarbox‐amidoadenosine** restores vascular barrier functions and reduces inflammatory injury in LPS‐induced ALI mice.^141^ These results support that adenosine receptor activation may offer a novel therapeutic approach for the management of ALI/ARDS (Figure 5C and Table 3).

### 4.4 Therapeutic agents targeting neuropeptides

VIP is a neuropeptide with multiple immunomodulatory effects. Lentivirus‐mediated **VIP** addition has been reported to attenuate ALI in LPS‐induced mouse models via inhibiting reactive oxygen species generation, NLRP3 inflammasome activation, and pro‐inflammatory IL‐17A expression in macrophages.^93^, ^94^ Sensory C‐type neurons‐derived α‐CGRP is one of the most abundant neuropeptides in the lung and possesses modulatory effects on immune functions. In an ALI rat model, LPS installation increases α‐CGRP level but reduces α‐CGRP receptor expression in the lung. Furthermore, exogenous **α‐CGRP** improves oxygenation and lung injury‐related index in ALI rats, which is associated with the upregulation of the transcription factor ICER (inducible cyclic adenosine monophosphate early repressor).^95^ Mechanistically, CGRP modulates macrophage polarization and inhibits inflammatory response in murine macrophages stimulated by LPS.^142^ Moreover, CGRP receptor antagonist CGRP8‐37 exacerbates LPS‐induced lung tissue damage in a rat model, accompanied by excessive pro‐inflammatory cytokines expression, as well as decreased levels of aquaporin‐1 and −5.^97^ Controversially, some scholars believe that CGRP is a mediator of neurogenic inflammatory response in several lung diseases caused by airway noxious stimuli. In an ovine ALI model of combined burn and smoke inhalation, pretreatment with a specific CGRP receptor antagonist **BIBN4096BS** attenuates early airway hyperemia, transvascular fluid flux and abnormalities in respiratory gas exchange.^96^ Accordingly, regulating pulmonary CGRP may become a potential therapeutic strategy for ALI/ARDS, but more studies are needed to clarify the concrete etiology when considering the neuropeptide.

Inhaled **leytragin**, an agonist of the *δ*‐opioid receptor, is demonstrated to inhibit HMGB1 secretion in LPS‐induced ALI of mice by preventing hyperacetylation at lysine residues and promoting sirtuin 1 to deacetylate HMGB1.^143^ Kinins are critical pro‐inflammatory peptides and act on B1 and B2 receptors. Treatment with B1 receptor antagonist **BI113823** significantly mitigates LPS‐challenged direct lung injury and sepsis‐induced pulmonary inflammatory response, as well as improves survival after severe polymicrobial sepsis.^144^ In an ALI mouse model, the bradykinin B1 receptor expression elevates upon LPS inhalation; whereas, posttreatment with its antagonist **R‐954** prevents cytokines/chemokines expression, leukocyte infiltration and protein leakage in the BALF, and decreases the airway hyperreactivity.^145^ Altogether, the data implicate that the kinin system, acting through the B1 receptor, participates in the pathogenesis and development of ALI (Figure 5D and Table 4).

### 4.5 Agents acting on the RAS signalling pathway

Studies have declared that RAS plays a vital and bidirectional role in multiple diseases. In the animal model, LPS or hyperoxia exposure increases the Ang II level and activates the RAS, leading to the occurrence and progression of ALI.^146^, ^147^ Vitamin D exerts anti‐inflammatory and anti‐fibrotic effects in various pulmonary disorders. A vitamin D agonist, **calcitriol**, is proved to be beneficial against ALI by muffling LPS‐induced lung permeability, which may be at least partially attributed to the expression balance of RAS members.^148^ In fact, subcutaneous Ang II infusion alone can establish the ALI mouse model by pathological identification; **IL‐22** administration shows protective effects on lung edema, inflammatory cell infiltration and vascular endothelial barrier damage in the mice by promoting nuclear translocation of STAT3.^149^ The biological activity of Ang II hinges on its interaction with distinct ATRs, and the pathological effect is mainly achieved by activating AT1R. Researchers have reported that endogenous Ang II aggravates pathogenetic conditions in ALI rats via AT1R, since AT1R antagonist **losartan** or **ZD7155** prevents inflammatory NF‐κB activation and pneumocytic apoptosis, and improves the alveolar fluid clearance.^146^, ^150^, ^151^ **Fraxinol**, a simple coumarin compound, can rebalance the expression between Ang II‐AT1R and Ang (1‐7)‐Mas axis in the presence of LPS, thereby exerting anti‐inflammatory and anti‐apoptotic actions both in ALI mice and LPS‐stimulated macrophages.^152^ Comparatively, AT2R mediates the opposite effect of AT1R and is able to abrogate Ang II‐AT1R axis‐induced excessive inflammatory response and oxidative stress. Compound 21 is a highly selective AT2R agonist and shows superior efficacy both in pulmonary fibrosis and novel coronavirus pneumonia. Our most recent study verifies that **Compound 21** alleviates acute inflammation and tissue damage in the lungs of LPS‐challenged ALI mice via reprogramming macrophage function.^153^

RAS activation can also protect against the development of ALI. **Lipoxin A4**, a product of arachidonic acid metabolism, has been found to protect ALI mice from lung damage via upregulating the ACE2‐Ang (1‐7)‐Mas receptor axis in the lung.^154^ Besides, **Ang (1‐7)** or its analogue **AVE0991** attenuates the key features of ALI, such as neutrophil infiltration, lung edema and pulmonary vascular resistance caused by a ventilator, acid aspiration or oleic acid infusion.^155^, ^156^ ACE2 is a critical negative regulator of RAS and exhibits beneficial properties on severe ALI caused by various etiologies.^157^, ^158^ For instance, recombinant **ACE2** remarkably reverses SARS‐CoV‐2 spike receptor‐binding domain protein‐induced ALI by directly cleaving AngI/AngII, further curbing NOX1/2 expression and their mediated inflammation and oxidative stress.^159^ Recombinant B38‐CAP is a bacteria‐derived ACE2‐like carboxypeptidase and is more efficient than recombinant human ACE2. In the abdominal sepsis‐ or acid aspiration‐stimulated ALI model, **B38‐CAP** degrades lung Ang II to Ang (1‐7), leading to the downregulated cytokine generation, decreased inflammatory injury and improved survival rate of the mice.^160^ In addition, B38‐CAP also shows beneficial efficacy in SARS‐CoV‐2‐infected hamsters or human ACE2‐transgenic mice.^161^ Treatment of ACE2 activator **resorcinolnaphthalein** or Ang (1‐7) reduces the severity of LPS‐induced ALI and pyroptosis, while AngII, ACE2 inhibitor MLN‐4760 or Mas inhibitor A779 significantly exaggerates them, suggesting the protective role of ACE2/Ang (1‐7)/Mas axis in the pyroptosis of ALI model.^162^

Several traditional Chinese medicines can also protect against ALI through RAS modulation. In an ALI rat model, paraquat‑augmented airway inflammation, vascular leakage, and tissue damage are attenuated by **tanshinone IIA** (also known as danshen), an active compound isolated from Salvia miltiorrhizae Bunge, via the increased expression of ACE2 and Ang (1‐7).^163^ Another example is **Sini decoction**, which has been reported to ameliorate sepsis‐ or E. coli‐induced lung injury in the mouse model via activating the ACE2‐Ang (1‐7)‐Mas axis to exert the anti‐inflammatory effect.^164^, ^165^ **Osthole**, a natural coumarin extracted from the fruit of Cnidium monnieri (L.) Cusson, is found to exhibit beneficial efficacy on LPS‐challenged ALI mice. Pretreatment with osthole reduces inflammatory mediators secretion, pulmonary vascular leakage, as well as mortality in mice with severe lung damage. Furthermore, osthole markedly prevents the decreased expression of ACE2‐Ang (1‐7) in the lung of ALI mice, while ACE2 inhibitor blocks the protective effects, implying the potential role of RAS in the anti‐inflammatory activity of osthole in ALI therapy.^166^ Collectively, the researchers provide evidence that drugs modulating RAS activation may become a conceptually new strategy for the clinical therapy of ALI with different etiologies, including the 2019 novel coronavirus (Figure 5E and Table 5).

## 5 CONCLUDING REMARKS

**FIGURE 6:**
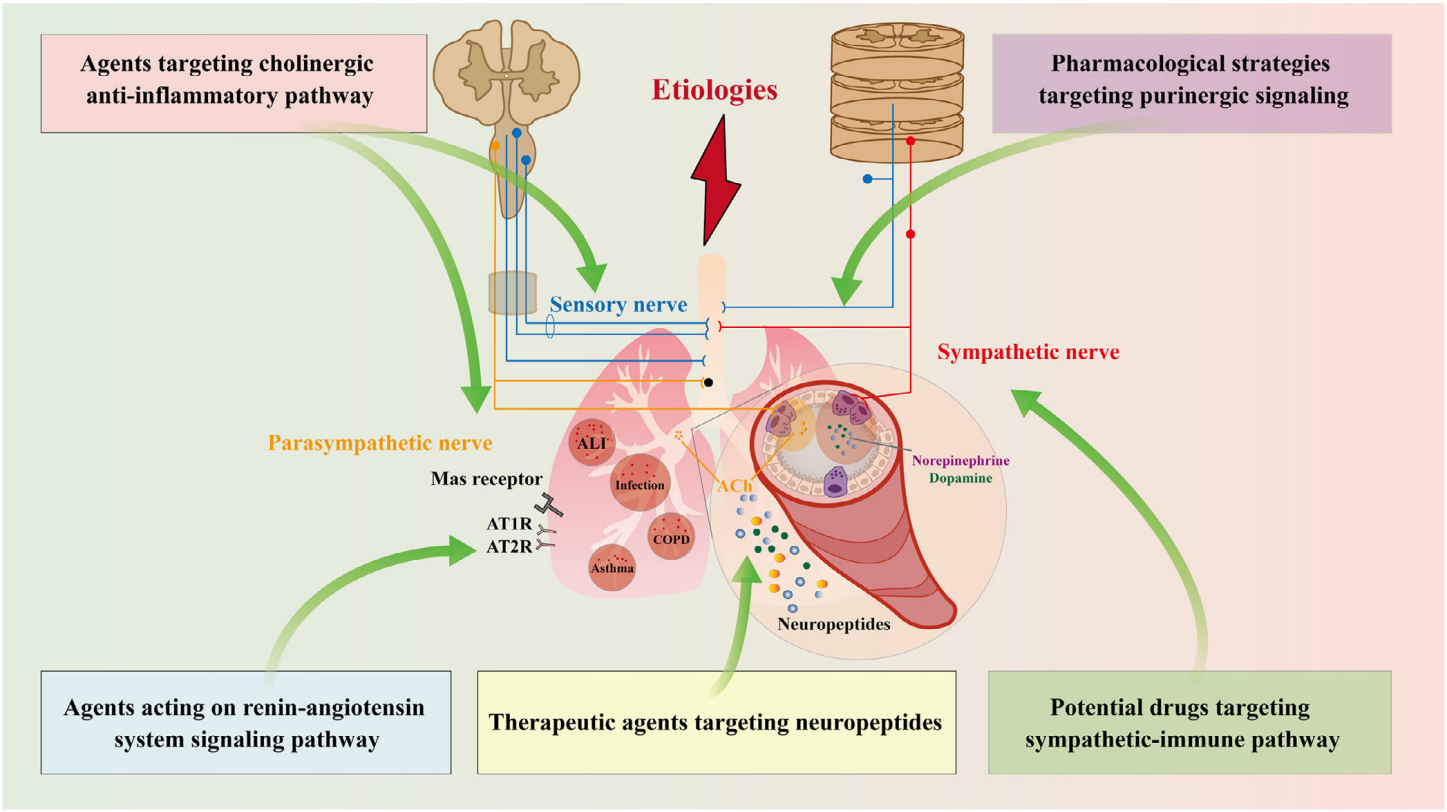
Promising neuroimmune targets for pulmonary diseases. AT1R, angiotensin II type 1 receptor; AT2R, angiotensin II type 2 receptor; ACh, acetylcholine; ALI, acute lung injury; COPD, chronic pulmonary obstructive disease.

Building on a series of studies performed decades ago, the neuroimmune crosstalk has become a hot research topic in the field of both neurobiology and immunology. The positive feedback between the immune and neuronal systems in lung tissues may be an amplifying mechanism to induce potent inflammatory reactions, which appear to cause health problems, such as pulmonary infection, asthma, COPD and ALI/ARDS. Therefore, the modulation of neuroimmune interaction is a promising therapeutic target for these diseases. To expand the research field in the coming future, it is necessary to investigate the influence of the peripheral nervous system and its neurotransmitters or neuropeptides on the pulmonary immune microenvironment, as well as summarize potential drugs targeting neuroimmune crosstalk in lung inflammatory diseases represented by ALI/ARDS (Figure 6).

Although these strategies have been widely accepted experimentally, very few have entered clinical trials to date, and none have been approved for clinical practice. Lessons have been drawn from these failures. First, the efficacy, stability, and safety of the therapeutic agents, and whether they would impair local or systemic immunity should be taken into account in future clinical development. More significantly, since danger signals or infection pathogens frequently activate multiple neuroimmune pathways, and single‐targeted drugs failed in clinical trials; developing more potent remedies acting through multiple peripheral nervous system signallings, or combined with other anti‐inflammatory therapeutics, may bring new hope for the treatment of ALI. Due to its heterogeneity, full consideration should be given to the specific type of ALI when applying a certain drug in order to avoid ineffective or even counterproductive effects. Besides, many ALI patients with underlying diseases already have an altered neuroimmune pattern, which may render previously effective drugs ineffective. For example, patients combined with COPD are likely to develop an enhanced cholinergic innervation, while patients suffering from chronic heart failure tend to have an overactivated RAS system. On this issue, choosing drugs with good targetability and minimal side effects or combined pharmacotherapy seems to be a better choice.

Furthermore, with the rapid progress of life sciences and biotechnology, new techniques could be used to seek a breakthrough. For instance, single‐cell RNA sequencing can be applied to identify novel biomarkers and cell types, as well as explore the influence of neuroimmune crosstalk on cellular heterogeneity in ALI. CRISPR‐CAS9 gene editing technology could be utilized to modify the expressions of neurotransmitters, neuropeptides and their receptors in specific cells, thus providing more precise tools to develop novel gene‐editing drugs. Besides, due to the size advantage, nano‐devices have better bio‐distribution and could be functionalized to meet the desired pharmacokinetic and pharmacodynamic profiles. Therefore, nanomedicine and nano‐delivery systems will shine the way to promote the clinical application of such a promising strategy in the near future.

## AUTHOR CONTRIBUTIONS

**Di Wu**: Investigation and writing—original draft. **Ximing Liao**: Investigation and visualization. **Jing Gao**: Visualization. **Yixuan Gao**: Validation and writing—review & editing. **Qiang Li**: Conceptualization and funding acquisition. **Wei Gao**: Project administration; funding acquisition and writing—review & editing.

## CONFLICT OF INTEREST STATEMENT

The authors declare no conflict of interest.

## FUNDING INFORMATION

This work was supported by the National Natural Science Foundation of China (grant number: 82000086) for Wei Gao, and the Top‐level Clinical Discipline Project of Shanghai Pudong (grant number: PWYgf2021‐05), National Natural Science Foundation of China (grant numbers: 82070086 and 82270116) for Qiang Li.

## Data Availability

The data that support the findings of this study are available from the corresponding author upon reasonable request.
